# Family Ties and Well-Being: The Role of Support Across Generations

**DOI:** 10.1093/geroni/igaf122.3295

**Published:** 2025-12-31

**Authors:** Yunann Lin, Hui-Ching Weng, Wang Chih-Liang

**Affiliations:** National Chung Cheng University, Minxiong, Chiayi, Taiwan (Republic of China); National Cheng Kung University, Tainan City, Tainan, Taiwan (Republic of China); National Cheng Kung University, Tainan City, Tainan, Taiwan (Republic of China)

## Abstract

Due to aging impact on traditional family structure, exploring their diverse needs is essential for older adult physical and mental health. Thereby, this study aimed to compare the intergenerational relationship to subjective well-being from both parents and children aspect in the baby boomer and generation X. This longitudinal study analyzed data collected from the 2016 Taiwan Social Change Survey, consisting of demographics, self-rated health, subjective well-being, family harmony, family conflict, financial, household, and emotional support. Participants were categorized into two cohorts: the baby boomer generation (1946-1964), and generation X (1965-1980). Logistic regression is adopted to analysis intergenerational associations. After adjusting gender, marriage, self-rated health, income, providing emotional support (p = 0.028) to parents increase subjective well-being in baby boomer generation for generation X. The baby boomer generation’s subjective well-being was positively associated with providing financial support (p < 0.001) to parents and receiving financial (p = 0.011) and household (p = 0.034) support from children. Additionally, family harmony with parents was positively associated with subjective well-being in baby boomer (p = 0.001) and generation X (p = 0.027); whereas family conflict with parents was negatively associated with subjective well-being in baby boomer (p = 0.04) and generation X (p = 0.004). In conclusion, intergenerational relationship is more significant to subjective well-being in baby boomer than generation X. Baby boomer generation concern providing and receiving support from parent and children, especially in financial aspect; while generation X value parent harmony more than family support.

